# Patients Unmet Needs in Chronic Rhinosinusitis With Nasal Polyps Care: A Patient Advisory Board Statement of EUFOREA

**DOI:** 10.3389/falgy.2021.761388

**Published:** 2021-10-29

**Authors:** N. Claeys, M. T. Teeling, P. Legrand, M. Poppe, P. Verschueren, L. De Prins, L. Cools, L. Cypers, W. J. Fokkens, C. Hopkins, P. W. Hellings

**Affiliations:** ^1^Patient Advisory Board of the European Forum for Research and Education in Allergy and Airway Diseases, Brussels, Belgium; ^2^Patient Liaison Officers of the European Forum for Research and Education in Allergy and Airway Diseases, Brussels, Belgium; ^3^The European Forum for Research and Education in Allergy and Airway Diseases Scientific Expert Team Members, Brussels, Belgium; ^4^Department of Otorhinolaryngology, Head & Neck Surgery, Academic Medical Center (AMC), Amsterdam, Netherlands; ^5^Guy's and St Thomas' Hospital, London Bridge Hospital, London, United Kingdom; ^6^Department of Otorhinolaryngology, Head and Neck Surgery, Universitair Ziekenhuis Leuven, Leuven, Belgium; ^7^Department of Otorhinolaryngology, University of Ghent, Ghent, Belgium

**Keywords:** nasal polyps, oral corticosteroids, quality of life, unmet needs, chronic rhinosinusitis

## Abstract

**Background:** European patients with chronic rhinosinusitis with nasal polyps (CRSwNP) have had only limited occasions to unite to have their voices heard, hence missing the opportunity to contribute to the improvement of CRSwNP care.

**Aims:** To identify unmet needs in CRSwNP from the perspective of CRSwNP patients from the Patient Advisory Board (PAB) of the European Forum for Research and Education in Allergy and Airways diseases (EUFOREA).

**Methodology:** Semi-structured interviews were conducted individually with 15 European patients with CRSwNP and with a disease history of more than 2 years. Patients shared their burden of the disease and frustrations related to CRSwNP care, experiences with key pillars of current treatment options, shortcomings of the current care pathways and recommendations for improvement of care. A panel of 30 members of the Patient Advisory Board reviewed the interview report and provided further input during 2 virtual meetings.

**Results:** CRSwNP patients indicated the need for greater awareness from society and physicians of the disease burden with impact on social function and well-being. Along with a loss of ability to smell and the continuous presence of secretions in the nose, most patients reported poor sleep quality and psychological impact as the most bothersome symptoms. Patients' frustrations relate primarily to the underestimation of the disease burden, the lack of coordination of care and the limited treatment options available to them. Treatment options with oral corticosteroids and/or sinus surgery both have positive and negative aspects, including the lack of long-lasting efficacy. Better coordination of care, more patient-centered care, greater public awareness, increases in research on the disease mechanisms and better therapeutic options would be warmly welcomed by CRSwNP patients.

**Conclusions:** This statement of the EUFOREA Patient Advisory Board on CRSwNP provides novel insights on the underestimation of the burden of CRSwNP and shortcomings of current care. Multiple recommendations made by the patients can underpin action plans for implementation of better care for CRSwNP among all physicians treating patients with this disabling disease.

## Introduction

Chronic Rhinosinusitis with Nasal Polyps (CRSwNP) represents a chronic inflammatory condition of the nose and paranasal sinus cavities with major impact on well-being and social function which is greatest in the young adult to middle aged populations (1). With an estimated prevalence of 3%, CRSwNP represents a common health problem in the Western world (1, 2). Despite international evidence-based guidelines for treatment (EPOS2020), a substantial group of patients remain uncontrolled with recurrent needs of oral corticosteroids (OCS) and/or endoscopic sinus surgery (ESS) (3).

The significant economic and clinical burden of CRSwNP highlights the need for better treatment options and reorganization of the current care pathways. A recent Dutch study revealed that the annual direct and indirect costs per patient were € 1,501 and € 5,659, respectively (4). The high financial impact of the disease resulted from costs related to health care utilization, absenteeism and lost work productivity (4, 5). Patients suffering from CRSwNP experience symptoms of nasal obstruction, smell dysfunction with anosmia in a large proportion, continuous nasal discharge and facial pain (1). Besides the sino-nasal symptoms, CRSwNP is associated with an increased incidence of depression and social dysfunction (6). Existing literature has found the impact of CRSwNP on quality of life (QoL) to be comparable to other chronic diseases such as chronic obstructive pulmonary disease (COPD), congestive heart failure and diabetes (7, 8).

In contrast to asthma, respiratory allergies and atopic dermatitis (9–11), few international patient initiatives have been undertaken to bring the burden of disease and other relevant factors of CRSwNP to the attention of health policy makers, to the general public or to physicians. Although it is widely recognized that upper and lower airway diseases are interrelated with inflammation in part of the airways (12), limited international initiatives have been undertaken to highlight the patient view and impact of diseases in both upper and lower airways. To meet these major unmet needs in the respiratory field, the EUFOREA Patient Advisory Board (PAB) was launched in 2017. The board is composed of 30 European patients from 8 European nationalities who suffer a long disease journey with chronic upper and lower airways diseases for more than 2 years. Patients of the PAB are regularly asked by the EUFOREA board and expert team leaders to share patient views on the burden of disease and care pathways, to advise experts on novel guidelines for respiratory care, and to help define strategies for better care (13–16).

Few qualitative studies on the patients' experiences and perspectives of current management of CRSwNP have been published. These studies identified patients' frustrations with delayed referral, poor communication, inconsistency of advice, incorrect medication use, adherence to intranasal steroids and lack of recognition of the impact of CRS (17, 18). This EUFOREA initiative aims at raising the CRSwNP patients' voice on the disease burden and key pillars of CRSwNP care. This 'Unmet needs in CRSwNP care' is launched as a valuable project to have the patients' voice heard, and to reflect on the current care pathways in all aspects. Fulfilling our mission to ease the burden that CRS patients have to manage, is the ambition of this EUFOREA project.

## Materials and Methods

### Patient Selection and Procedures

Fifteen European patients with CRSwNP of the EUFOREA Patient Advisory Board (PAB) were randomly selected by PH from the 30 PAB members for being interviewed by LD in March 2021. The number of participants was predetermined. Patients were selectively recruited based on a wide range of characteristics such as age, gender, nationality, severity of disease, duration of disease and CRS management, and willingness to be interviewed. The diagnosis of CRSwNP had to be confirmed by a local Ear, Nose and Throat specialist prior to recruitment, with only secondary or tertiary care patients interviewed and participating in this initiative. A list of contact data was provided by the EUFOREA patient liaison officers LCy and LCo. Invitation emails were sent to patients to request their consent and participation in a 20 min. telephone interview on the impact of CRSwNP on their daily life.

In-depth and semi-structured one-on-one interviews were conducted in English, French or Dutch language in order to facilitate the inclusion of diverse demographic characteristics and to avoid the limiting factor of a language barrier. All interviews were carried out by one trained trilingual female clinician (LD, MD in training) who was not involved directly in the participants' care. The objective of the study was explained to the participants. Patient characteristics including age, gender, nationality, symptom duration, presence of comorbid asthma, severity of CRS symptoms on a visual analog scale ranging from 0 (no symptoms) to 10 (worst thinkable symptoms) and current treatment were questioned.

An open-ended questionnaire was designed by PH and approved by the EUFOREA experts CH and WF. This predefined template was used as a guide to generate discussions and to document all aspects of CRSwNP that affected the participants. Field notes were made during the interview to provide context. Participants were asked to reply to the following predefined questions:

- What is the major burden of CRSwNP?
- What are your major frustrations regarding CRSwNP care?
- What do you consider the benefits and shortcomings of OCS?
- What do you consider the benefits and shortcomings of ESS?
- What are the shortcomings of the current CRSwNP care pathways?
- What suggestions do you have for overall improvement of care for CRSwNP?

Additional comments, corrections, suggestions and approval was provided during two virtual meetings with a review panel of 30 patients of the PAB. This review was performed to validate the findings from the interviews. No repeat interviews were carried out.

### Analysis

The interview sessions were audio-recorded and transcribed. Transcribed recordings were managed using oTranscribe software. All transcripts were analyzed qualitatively by one researcher and the qualitative analysis was reviewed by multiple participants. Frequently occurring and important statements were highlighted and categorized for similarities in content. Themes were identified in parallel with the interview questions and determined according to the responses collected. Significant direct quotes were noted separately to illustrate general opinions. Clear summary figures were designed based on the reported strengths, weaknesses, shortcomings, and suggestions for current care pathways with the aim of concisely presenting the unmet needs from the patient perspective.

### Ethical Considerations

All patients have provided written consent for participation in this analysis, and those listed as co-author have been interviewed and explicitly approved to be listed as a member of this PAB initiative via written consent.

## Results

### Interview Specifics

This study recruited 15 patients to participate in a one-on-one interview in April 2021. Phone calls ranged in length from 10 to 50 min with a mean duration of 17 min 18 sec. All interviews were conducted in March 2021.

### Participant Characteristics

The mean age of participants was 52 years and 53% were men. Estimated history of CRS symptoms ranged between 2 and 52 years with a mean of 22 years. Nearly two out of three study patients suffered from comorbid asthma. Participants rated the severity of their CRS disease by an average of 6/10. The selected patients represented 7 different European nationalities. Baseline characteristics of the patients included are presented in Table 1.

**Table 1:** Patient characteristics.

	**Total (*n* = 15)**
Age (mean, range, in years)	52 (18–69)
Gender	
Female	7
Male	8
Diagnosis period (mean, range, in years)	22 (2–52)
Comorbid asthma	
Yes	9
No	6
Severity of CRS (mean, range, on scale 0–10)	6 (2–8)
Current treatment	
Nasal treatment	14
Oral treatment	10
Inhaled treatment	9
Previous sinus surgery	
Yes	14
No	1
Biological	
Yes	2
No	13
Nationality	
Belgian	8
Dutch	2
German	1
Swedish	1
Greek	1
Luxembourgish	1
Danish	1

Various themes were identified such as major burden and frustration of CRSwNP, experiences with CRPwNP treatments and the role of the EUFOREA Patient advisory board.

### CRSwNP in Daily Life—Major Burden and Frustrations

Most patients experienced a major impact of CRSwNP on their daily lives as a result of a wide variety of disease symptoms. Overall, participants were incredibly frustrated about the underestimation of the burden of disease, with the perception of others often being that.

“*I think there still is not enough knowledge in primary care. CRSwNP is still too often compared to a common cold or a small headache.”*

As many as 12 of the 15 interviewees acknowledged living with a lack of smell (and taste) capacity. This olfactory dysfunction limited their pleasure of sharing a dinner with family, colleagues or friends. In addition, it placed patients in awkward and potentially dangerous situations.

“*I don't notice the smell of a grandchild's dirty diaper.” “Living with no sense of taste and smell is like watching TV in black and white in the 21st century*. […] *You miss a lot of impressions of the surroundings. You don't get any input.”*

“*When I go to friends for dinner, I often don't taste the food. I don't dare to say this then, because the problem is so difficult to explain and no one understands it.”*

Besides smell reduction, participants reported suffering from other typical symptoms such as nasal obstruction/blockage, rhinorrhea/postnasal drip, sneezing, hearing impairment, teary eyes, bad breath, and facial pain/pressure. Participants, especially those with comorbid asthma, felt embarrassed because these clinical symptoms may be mistaken for symptoms of COVID-19 (19). Some patients also mentioned that they avoided drinking alcohol in order to escape an increase in nasal symptoms. The issues mentioned above were identified as the cause of reduced social contact and social embarrassment.

“*I am never able to leave the house without handkerchiefs.”*

“*It seems like I'm continuously out of breath.”*

“*I have to blow my nose all the time, this made customers at work think I had COVID-19. That was very confronting.”*

“*Nobody wants to go to a party when they have a cold.”*

“*I cannot drink half a beer. It makes me blow my nose all the time and I can't talk anymore.”*

“*I feel ashamed of my nasal voice.”*

Overall, participants agreed with the remarkable psychological impact of CRSwNP. The recurrent upper respiratory tract infections in addition to the continuous presence of physical and mental symptoms caused stress and a depressed mood. One out of five participants had a physician-diagnosed depression.

“*Imagine having a cold for 20 years, this breaks you down slowly*. […] *You never get a break, it never goes away.”*

“*I'm afraid my children will have to go through the same thing.”*

“*I can never be just normal. I had to find another way of living: a steady rhythm, spreading the load, learning how to dose, going to bed on time…”*

The burden of poor sleep quality was mentioned numerous times. While some patients associated nasal obstruction and snoring with their sleep dysfunction, others felt that post-nasal drip was the leading cause. Daytime somnolence and increased fatigue had harmful effects on both individual productivity, social function and work performance.

“*I can't focus on anything for 100%*. […] *I feel really tired when I wake up, that's the worst time of the day. That's why I always look and feel exhausted.”*

“*I feel embarrassed to sleep among others because of my snoring.”*

### Experiences With Current CRSwNP Treatment Options—OCS and ESS

**Figure 1:**
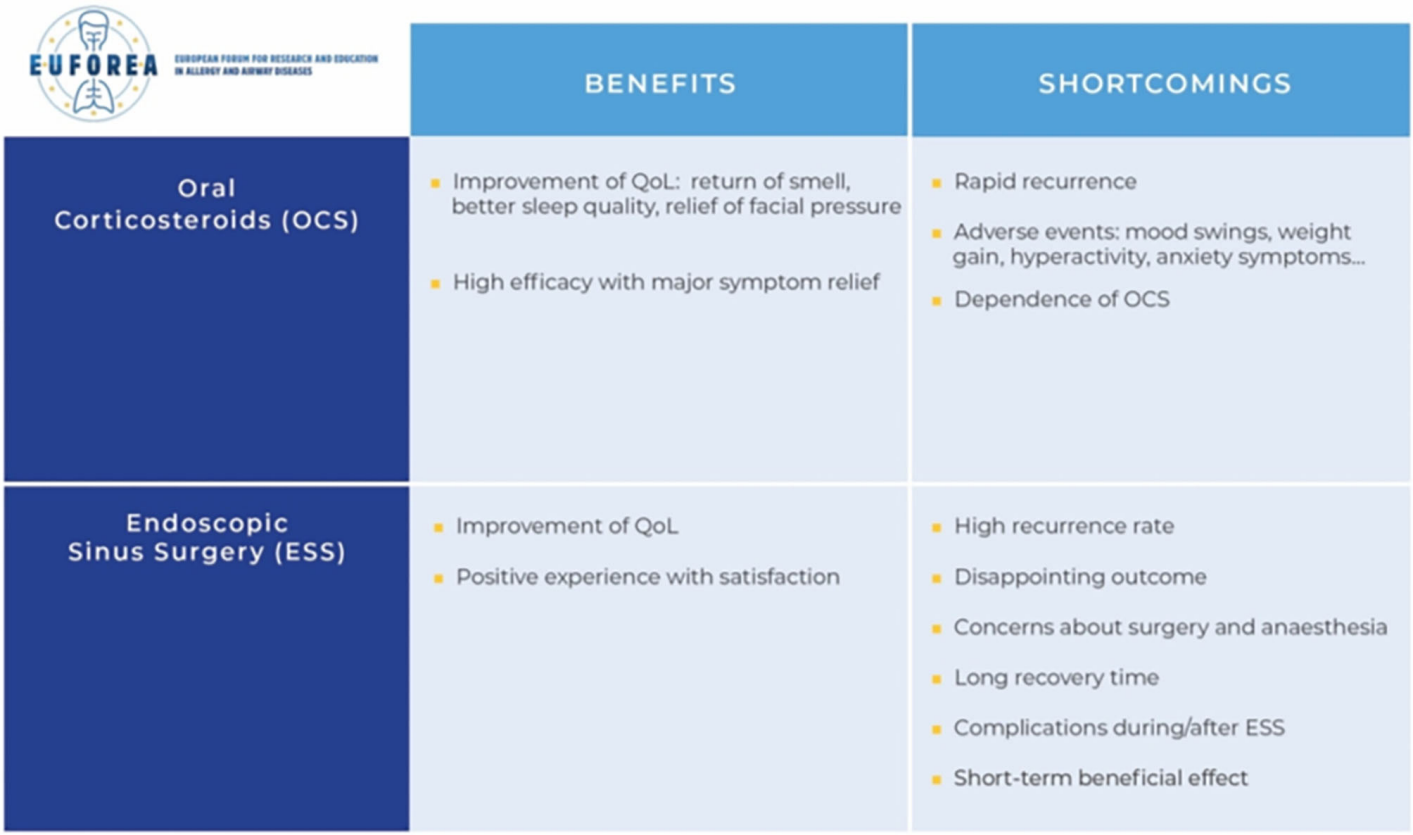
Strengths and weaknesses of the current options with OCS and ESS.

Figure 1 shows an overview of the strengths and shortcomings of OCS and ESS. When asked about treatment related factors, several patients described the adverse effects of their treatments as disabling.

“*I am often more sick from my medication than from my symptoms.”*

A majority of patients felt frustrated about the lack of an effective treatment and emphasized the need for a treatment that targeted the cause of their disease.

Some participants reported the underestimation of the importance of alternatives to OCS such as nasal rinses with Saline.

“*I haven't discovered anything that works yet, nothing helps.”*

“*Conventional medicine fails, it has no answer. I started to look outside conventional medicine for tools to improve my quality of life. E.g., kinesiologist, yoga, mindfulness osteopathy…”*

“*My current treatment does not target the cause of the disease, it just obscures my symptoms.”*

### OCS

One out of three respondents regarded the use of oral corticosteroids as effective. This group regained their ability to smell and taste, was relieved from facial pressure, headaches, nasal drainage, and experienced a significant improvement of sleep quality. However, others mentioned the low efficacy of this medical treatment, and some patients experienced a reduced effect over time. Multiple specific side effects were described including hyperactivity, insomnia, swelling of the face, mood swings, weight gain, reduced bone mineral density, and anxiety symptoms. Patients regretted the overuse and prescription of long-term OCS. The risk of addiction to OCS given the good mood was mentioned several times.

“*The OCS only worked for a couple of weeks.”*

“*It's like choosing between the plague and cholera. When I take OCS, I am relieved of my nasal secretions and I regain my smell. The downside is that I gain weight and I cannot sleep well anymore because it makes me hyperactive. I need to weigh the pros and cons against each other.”*

### ESS

Fourteen patients underwent sinus surgery, of which 8 have had more than one surgery. Only a few among them described the surgery itself as a positive experience. However, almost all patients experienced a significant improvement of quality of life afterwards.

“*Symptoms were as good as gone, I regained my smell function.”*

“*I still suffered from nasal blockage, but the periods were shorter.”*

The occasionally only temporary and unpredictable outcome in combination with the consequent need for lifelong surgeries was described as most disadvantageous, followed by the often long recovery time/loss of workdays. Patients acknowledged feeling concerned about the possible complications of sinus surgery. Only a minority of patients actually experienced complications of surgery and/or general anesthesia.

“*I was very disappointed about the result of the operation.”*

“*I have to take 14 days off each time after surgery. Then I'm not able to work.”*

*Patients' view of the current CRSwNP care pathway–Major shortcomings and suggestions*

### Major Shortcomings

Patients considered the lack of coordination in care as a major shortcoming in the current health care organization. The inconsistency between specialists in how patients are managed was a source of frustration. Many patients suffered from impactful comorbidities of which asthma was the most common. Among these patients, few felt comfortable about the care they received for their comorbidities; the majority reported a lack of attention to comorbidities.

“*I have already seen over six specialists. Every doctor has his own vision about the treatment of the disease, doctors contradict each other. They should be more consistent among each other.”*

Some patients admitted they felt the search for appropriate help was long and tough, whereas-others felt that their referral from the GP to the specialist proceeded smoothly. Several patients expressed having a feeling that there is a lack of knowledge in care. According to the patients, there is a lack of personalized care and patient participation and they expressed a desire to feel more independent.

“*Conventional medicine searches for diagnosis and treats them without looking at the patient itself. Classic medicine does not search enough for the cause.”*

“*It feels like I have more knowledge about myself/my disease, than the doctor has.”*

“*Doctors do not pay attention to things that are not scientifically proven. I believe that a change of lifestyle and nutritional supplements can help to reduce my symptoms.”*

“*I find it tiresome that I have to make an appointment for new prescriptions every time, while I have been taking this medication for 10 years already. This requires a lot of time and money.”*

Patients also faced difficulties in being taken seriously by healthcare professionals. They perceived an underestimation of the social- and psychological aspects of CRSwNP as well as a general lack of awareness about the disease. Some patients pointed out the financial impact of CRS, due to the ongoing need for multiple medicines and the impact on employment.

“*Please don't understand me wrong… cancer is a very serious illness, it's logic that a huge budget is released for this. I know you don't die from CRS, but this disease does have a huge impact on my life… day in day out. It would be nice if we received some attention and financial resources too.”*

As cited above, patients reported the lack of reimbursed access to new treatments. In particular, the unavailability of new treatment options including biologicals such as Dupilumab was mentioned.

“*I experienced the good effects of Dupilumab during a clinical trial. Now I know there is a solution, but I don't have access to it. That is really frustrating.”*

“*I'm afraid my children will also have CRS, I hope that by then there will be a treatment that can cure the disease.”*

Patients reported the inconsistency within European countries as frustrating. Some of them needed to travel to other countries to purchase the medication they need.

“*Flixonase nasules are not available in Belgium. I need to buy them in the Netherlands, which is a long journey every time and they are not refunded.”*

“*I explored the European market to find the best price for Flixonase nasules. I order them in Spain.”*

### Suggestions on Future Care

According to patients, the pooling of expertise and conferences on the latest developments in the field of CRSwNP will be essential to share information and to provide the most appropriate care. Patients proposed training for GP's to ensure faster diagnosis and referral more accurately. Respondents reported the implementation of joint clinics as a good solution to optimize the approach of multimorbidities. In parallel, the mention of CRSwNP in asthma/pulmonology guidelines was proposed.

“*My GP, pulmonary specialist and ENT specialist must work together.”*

“*Doctors need to get in line with each other.”*

“*They need to organize extra courses for specialists and GPs to inform them about the latest treatment options.”*

Participants highlighted the benefit of proper training on correct medication use as well as the implementation of smartphone applications into clinical practice.

“*Can't they make a device to link my symptom recording and medication lists to my medical file?”*

Patients emphasized an individual approach in the CRS care pathway, and some even begged to have more attention paid to the diversity of patients. Discovering which environmental factors trigger symptoms was a request of multiple patients.

“*Specialists should experiment with the different treatment options to see what works the best for each individual.”*

Another patient-reported idea was the development of specified psychological services to improve the attention for mental health. Patients expressed the demand for research on new treatment options. They hoped to see the availability and reimbursement of novel therapies increase in the future.

“*Please develop a therapy against my runny nose. There is no treatment on the market that relieves me from that.”*

“*There's no treatment that can control my symptoms.”*

### Role of EUFOREA Patient Advisory Board

Patients mentioned that EUFOREA can raise public awareness for the CRS burden that many people still underestimate even in 2021. It was reported by participants that more attention is needed for the impact of CRS on school- and work performance.

“*I repeated a grade in high school because of multiple infections and a sinus surgery that year. Teachers need to know that you can't control this. You don't do this on purpose.”*

“*I would like to maximize the outreach of this initiative, beyond the local group of EUFOREA.”*

According to the PAB patients, EUFOREA can play a more important role in the advocacy to health policy makers. Patients believed that EUFOREA can make the patients' voices heard at a policy level. They highlighted the importance of putting pressure on the approval of biologicals as well as on the release of budget for further research. Patients believed that EUFOREA can, as a leading organization, initiate the development of European guidelines on the reimbursement and availability of treatment options. From the patients-view, EUFOREA was the perfect medium to focus on the empowerment of patients through education.

“*EUFOREA could inform patients about the possible side effects of their medication.”*

“*Maybe the PAB could draw up a list with tips and tricks from patients to patients e.g., alcohol avoidance, patient experiences with treatments…Inside information about the possible risk factors and triggers would be very valuable.”*

**Figure 2:**
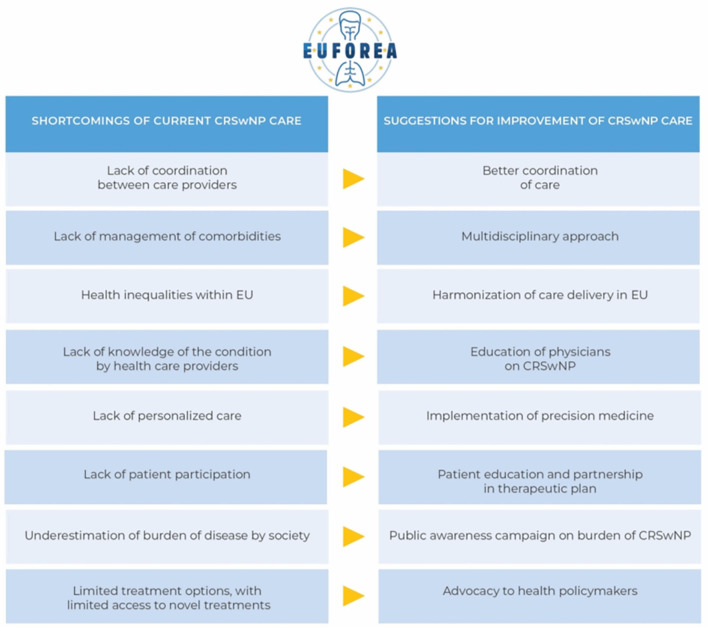
Shortcomings and suggestions for improvement of current care pathways.

A summary of the shortcomings and suggestions for improvement of current care pathways is shown in Figure 2.

## Discussion

CRSwNP is a common chronic disease with a major impact on the daily lives of patients (2). This initiative of the PAB of EUFOREA provides unique insights into the burden of CRSwNP, patients' views on the strengths and shortcomings of the current care pathways and patients' priorities and preferences to overcome these unmet needs in future CRS management. CRS patients reported some very important insights in addition to the classic CRSwNP symptoms presented in previously reported studies (2, 18). They noted impaired sleep quality, along with mental dysfunction and reduced smell capacity, as the most frustrating aspects of the disease. Further typical CRSwNP symptoms include nasal congestion, nasal drainage and facial pressure (20).

Our findings highlighted coordination in care as one of the key unmet needs. A multidisciplinary approach, in particular the improvement of communication between healthcare professionals, the inclusion of CRSwNP in asthma/pulmonology guidelines and the introduction of joint clinics in hospitals, can help to foster a holistic and multidisciplinary patient approach (21). Management should be applied according to guidelines and thus according to generally accepted consensus on the best treatment for any specific patient (22). Residents of different countries in Europe do not enjoy the same rights and conditions regarding the availability and reimbursement of treatments. European homogenization on availability of medication, treatment options and plans according to guidelines may be a good step forward.

A more patient-centered approach is an additional factor that many patients are asking for. In accordance with previous reports, precision medicine and in particular personalized medicine and shared decision-making will increase satisfaction, therapy compliance and control of disease (23).

Supporting patients in better management of their health demands more insight and advice on environmental factors that aggravate the disease. A broad-minded view of physicians on life-style and preventive medicine would be appreciated, although the limited evidence to support such approaches must be acknowledged (1). In addition, an individual approach can identify and correctly manage the impactful symptoms and comorbidities from which participants suffer (21). Patients strive for more independence in disease management. This empowerment can be achieved both by implementing mobile health apps, organizing patient training on correct medication use and customizing the legislation regarding prescriptions for chronically ill patients (21, 24).

Another important finding was the underestimation of the burden of disease. The importance of the psycho-social aspect including mental health, sleep quality, and social function is often forgotten. The development of specified psychological services to teach patients how to cope with their disabling disease would be a great advance in health care. Public awareness can help to create a more empathetic environment (13).

Despite the many positive effects of sinus surgery and oral corticosteroids, there are many limitations including disappointing outcomes and adverse events associated with the 2 major pillars of current treatment (2, 3). The dependence on drugs and recurrent surgeries restrict patients in their freedom. In fact, the absence of causal therapies is a clear unmet need. Therefore, as indicated by patients, future research on deeper understanding of pathophysiological mechanisms and potential novel treatment options is needed.

EUFOREA represents a European forum to help overcome these variously experienced shortcomings. The organization is built to develop solutions to bridge the gap between guidelines and daily practice (13). EUFOREA's comprehensive website contains a wide range of educational material and evidence-based information about the symptoms, diagnosis and possible treatments of CRS (1). In the future, this website will be expanded to include information on prevention, environmental triggers, comorbidities of CRS, life-style advice and potential side effects of different medical/surgical treatments. Focus on prevention rather than treatment can reduce the financial burden that patients experience (23, 25). The organization of diverse events for both patients and health-care providers can be further expanded by EUFOREA (13). Themes cited by patients for this include trainings for patients on how to use their medication, testimonials from peers, campaigns to raise awareness on a public level, the education of GPs on how to speed up and optimize the referral process, tools for implementing the concept of precision medicine, and courses for specialists on the latest treatment options. EUFOREA has already developed a multitude of projects to address all these issues, but to increase the awareness of the EUFOREA organization itself remains a future ambition. Patients see EUFOREA as an important tool for raising awareness among all stakeholders and health policy makers. Raising the disease higher on the political agenda may release additional funding for research and may also expedite the process of approval and reimbursement of novel therapies.

A certain limitation of this study includes the participant selection. Participant recruitment based on voluntary involvement and membership of the PAB may imply that these patients have more interest in their disease. Nevertheless, except the fact that almost all patients have undergone surgery, the characteristics of this participant group were balanced and heterogeneous which is one of the strengths of this study. The diverse patients' unmet needs of CRSwNP reflect the major burden on patients' quality of life and care plans that are often inappropriate. Patients hope that joining forces with EUFOREA through this unique initiative can influence political discussions and raise public awareness. EUFOREA plays a leading role in bringing these valuable statements to health policy makers, implementing them into daily practice and thereby improving the quality of future health care.

## Conclusion

This statement of the EUFOREA PAB on CRSwNP provides novel insights into this underestimated and undertreated disease. Multiple recommendations made by patients can underpin action plans for implementation of better care for CRSwNP amongst all physicians treating patients with this disabling disease.

## Data Availability Statement

The original contributions presented in the study are included in the article/supplementary material, further inquiries can be directed to the corresponding author/s.

## Ethics Statement

The studies involving human participants were reviewed and approved by Ethische commissie onderzoek Universitair ziekenhuis/Katholieke universiteit Leuven. The patients/participants provided their written informed consent to participate in this study.

## Author Contributions

LD and PH have designed the questions and proposed these questions for approval and discussion by the PAB members. All authors have revised and approved the content and outcomes of the patient interviews.

## Funding

Funding was provided via an unrestricted grant to EUFOREA by Sanofi Genzyme and Regeneron.

## Conflict of Interest

The authors declare that the research was conducted in the absence of any commercial or financial relationships that could be construed as a potential conflict of interest.

## Publisher's Note

All claims expressed in this article are solely those of the authors and do not necessarily represent those of their affiliated organizations, or those of the publisher, the editors and the reviewers. Any product that may be evaluated in this article, or claim that may be made by its manufacturer, is not guaranteed or endorsed by the publisher.

## Abbreviations

CRSwNP: Chronic Rhinosinusitis with Nasal Polyps
ESS: Endoscopic Sinus Surgery
EUFOREA: European Forum for Research and Education in Allergy and Airways diseases
OCS: Oral Corticosteroids
PAB: Patient Advisory Board
QoL: Quality of life
GP: General practitioner.
