# Corrigendum: Patients Unmet Needs in Chronic Rhinosinusitis With Nasal Polyps Care: A Patient Advisory Board Statement of EUFOREA

**DOI:** 10.3389/falgy.2021.789425

**Published:** 2021-10-29

**Authors:** N. Claeys, M. T. Teeling, P. Legrand, M. Poppe, P. Verschueren, L. De Prins, L. Cools, L. Cypers, W. J. Fokkens, C. Hopkins, P. W. Hellings

**Affiliations:** ^1^Patient Advisory Board of the European Forum for Research and Education in Allergy and Airway Diseases, Brussels, Belgium; ^2^Patient Liaison Officers of the European Forum for Research and Education in Allergy and Airway Diseases, Brussels, Belgium; ^3^The European Forum for Research and Education in Allergy and Airway Diseases Scientific Expert Team Members, Brussels, Belgium; ^4^Department of Otorhinolaryngology, Head & Neck Surgery, Academic Medical Center (AMC), Amsterdam, Netherlands; ^5^Guy's and St Thomas' Hospital, London Bridge Hospital, London, United Kingdom; ^6^Department of Otorhinolaryngology, Head and Neck Surgery, Universitair Ziekenhuis Leuven, Leuven, Belgium; ^7^Department of Otorhinolaryngology, University of Ghent, Ghent, Belgium

**Keywords:** nasal polyps, oral corticosteroids, quality of life, unmet needs, chronic rhinosinusitis

In the original published article, there was an error in the Funding statement. The incorrect statement read “Funding was provided via an unrestricted grant to EUFOREA by Sanofi Genzyme.” The correct statement is “Funding was provided via an unrestricted grant to EUFOREA by Sanofi Genzyme and Regeneron.”

The authors apologize for this error and state that this does not change the scientific conclusions of the article in any way. The original article has been updated.

## Publisher's Note

All claims expressed in this article are solely those of the authors and do not necessarily represent those of their affiliated organizations, or those of the publisher, the editors and the reviewers. Any product that may be evaluated in this article, or claim that may be made by its manufacturer, is not guaranteed or endorsed by the publisher.

